# Kinect-based rapid movement training to improve balance recovery for stroke fall prevention: a randomized controlled trial

**DOI:** 10.1186/s12984-021-00922-3

**Published:** 2021-10-11

**Authors:** Melisa Junata, Kenneth Chik-Chi Cheng, Hok Sum Man, Charles Wai-Kin Lai, Yannie Oi-Yan Soo, Raymond Kai-Yu Tong

**Affiliations:** 1 Department of Biomedical Engineering, The Chinese University of Hong Kong, Hong Kong, SAR China; 2 Department of Sports Science and Physical Education, The Chinese University of Hong Kong, Hong Kong, SAR China; 3 Physiotherapy Department, Shatin Hospital, Hong Kong, SAR China; 4 Department of Medicine and Therapeutics, Prince of Wales Hospital, The Chinese University of Hong Kong, Hong Kong, SAR China

**Keywords:** Falls, Stroke rehabilitation, Slip and fall, Balance, Posture, Telerehabilitation

## Abstract

**Background:**

Falls are more prevalent in stroke survivors than age-matched healthy older adults because of their functional impairment. Rapid balance recovery reaction with adequate range-of-motion and fast response and movement time are crucial to minimize fall risk and prevent serious injurious falls when postural disturbances occur. A Kinect-based Rapid Movement Training (RMT) program was developed to provide real-time feedback to promote faster and larger arm reaching and leg stepping distances toward targets in 22 different directions.

**Objective:**

To evaluate the effectiveness of the interactive RMT and Conventional Balance Training (CBT) on chronic stroke survivors’ overall balance and balance recovery reaction.

**Methods:**

In this assessor-blinded randomized controlled trial, chronic stroke survivors were randomized to receive twenty training sessions (60-min each) of either RMT or CBT. Pre- and post-training assessments included clinical tests, as well as kinematic measurements and electromyography during simulated forward fall through a “lean-and-release” perturbation system.

**Results:**

Thirty participants were recruited (RMT = 16, CBT = 14). RMT led to significant improvement in balance control (Berg Balance Scale: pre = 49.13, post = 52.75; *P* = .001), gait control (Timed-Up-and-Go Test: pre = 14.66 s, post = 12.62 s; *P* = .011), and motor functions (Fugl-Meyer Assessment of Motor Recovery: pre = 60.63, post = 65.19; *P* = .015), which matched the effectiveness of CBT. Both groups preferred to use their non-paretic leg to take the initial step to restore stability, and their stepping leg’s rectus femoris reacted significantly faster post-training (*P* = .036).

**Conclusion:**

The RMT was as effective as conventional balance training to provide beneficial effects on chronic stroke survivors’ overall balance, motor function and improving balance recovery with faster muscle response.

*Trial registration:* The study was registered at Clinicaltrials.gov (https://clinicaltrials.gov/ct2/show/NCT03183635, NCT03183635) on 12 June 2017.

## Introduction

Telerehabilitation and interactive body motion detection technology using Kinect and Wii Fit have become more popular [1, 2]. The COVID-19 is a global pandemic started in the end of 2019 and is still ongoing at the preparation of this paper. Stroke survivors had limited access to the outpatient rehabilitation therapy during the COVID-19 pandemic. Telerehabilitation or computer-assisted training system using body motion detection technology can be applied for stroke rehabilitation during the COVID-19 pandemic by minimizing face-to-face interaction and the risk of infection. A recently published study in 2019 examined the effectiveness of an invention program which aimed to improve arm motor function in stroke survivors through 36 therapy sessions (70 min each) [1]. The program consisted of exercises, functional games, and stroke education. Their results showed home-based telerehabilitation was as effective as in-clinic rehabilitation and had significant improvements in arm motor function. Balance control and fall prevention are another important rehabilitation programs for stroke survivors. Healthcare professionals and stroke survivors are interested in the feasibility of using these computer-assisted training systems for balance training, and they would like to know the effectiveness when compared with conventional balance training (CBT) in the rehabilitation center.

Fall is more common in stroke survivors than age-matched healthy older adults [3]. Previous studies have reported that disturbances in muscle coordination [4] and between-limb synchronization [5], spasticity, and loss in dexterity from stroke [6] are the primary causes of falls [7] in adults with stroke, leading to an increased financial burden to the individual and the society [8]. To prevent falling and the severe injuries that may follow, it is vital to train the stroke survivors’ ability to maintain or recover their balance [9]. When experiencing postural disturbance, a person can perform a successful balance recovery by controlling the position and the motion of his/her body’s center-of-mass (COM) over the base-of-support (BOS) with mainly one of two strategies: fixed-support and change-in-support reactions. Fixed-support reactions rely on the generation of muscle torques from the ankle and/or hip joints to slow down or stop the movement of COM without changing the location and size of the BOS. In the change-in-support reactions, it involves rapid stepping and/or reaching-to-grasp a nearby object to increase the size of the BOS. Change-in support reactions can be very effective in providing the stabilizing force needed to restore postural balance when there is an unpredictable balance perturbation as they can arrest a much larger and faster COM motion [10]. Successful change-in-support balance recovery reactions require both fast response time (i.e. movement completion time) for rapid movement and adequate range-of-motion to prevent falls [7, 11]. These reactions are executed more rapidly than volitional limb movements but are often impaired in stroke survivors, researchers were investigated in reactive stepping related to falls in stroke rehabilitation [12].

Stroke survivors generally have a more prolonged reaction latency, slower movement speed, and less effective response to the external perturbation compared to non-stroke adults [13]. Specifically, it has been found that stroke survivors have delayed paretic muscle onset latencies compared to healthy older adults [14] and their own non-paretic limb in simulated falls. Previous studies have found that agility and perturbation training can effectively improve stroke survivors’ balance recovery reaction latency. Other than improving step reaction time, training can lower the prevalence of the stroke survivor’s fall incidents compared to conventional balance training (CBT) [14]. Similarly, Reactive Balance Training with fast and functional movements are beneficial for persons with fall risk [15, 16]. As a result, task-specific training could train faster response and movement times in chronic stroke survivors, and it is one of the potential training principles in fall prevention.

Balance exercises are typically prescribed by physiotherapists to chronic stroke survivors in clinical settings to reduce their fall risks [17, 18]. These exercises are usually emphasized in improving the range-of-motion and muscle strength in the paretic limbs to restore their physical ability. Moreover, similar to other stroke functional rehabilitation programs, the balance exercises require intensive and repetitive task-specific practice [19, 20]. Enormous social, emotional, health, and financial burden and strains are laid on the informal caregivers (spouse, children, siblings, or other family relations) as these caregivers are often called upon to administering exercises at home or sending the stroke survivors to rehabilitation centers [21]. Healthcare professionals (e.g., physiotherapists and occupational therapists), particularly those within the public healthcare system, may experience burn-out and stress, as they are often overwhelmed with the workload of stroke and other types of rehabilitations [22]. Telerehabilitation and interactive body motion detection technology using Kinect are having the potential to reduce the further increase of workload to the healthcare professionals. The system can provide guidance to users directly during the training and have training reports after the intervention for the clinical professional to follow up with each case.

Our study aimed to develop a Kinect-based guided balance training system for stroke rehabilitation and has the potential for home-based telerehabilitation. With the advancement of depth camera and body tracking technology and algorithm to identify body joint movements in 3D space (e.g. Realsense, Microsoft Kinect and Microsoft Azure Kinect, Wii Fit), many rehabilitation centers have adapted these affordable 3D motion capture technology to enhance their clinical practice [2, 13]. These technologies are being used to make rehabilitation methods more personalized, easy-to-use, and interactive than repetitive and high-intensity training in the traditional setting. Kinect-based physical therapy systems from a few established companies have obtained FDA 510(k) clearance (i.e. the medical device is safe and effective, and the device can be marketed for clinical use and is substantially equivalent to a legally marketed device), and previous studies have shown that Kinect and other technology-based systems could be used for both the training and assessment of reaching, gait analysis, stepping and balance [25–27]. They showed that technology-based rehabilitation was a suitable method for fall risk assessment and training to minimize fall risk. Previous studies utilized depth and tracking camera technology and virtual reality for balance and gait analysis purposes; however, their effectiveness has not been assessed for intensive balance training on chronic stroke survivors in a large-scale study nor in a randomised controlled trial [25–37]. Furthermore, previous studies did not assess its feasibility to improve the biomechanics and effectiveness of balance recovery reaction [10].

This study proposed a novel, task-specific Rapid Movement Training (RMT) program that would require stroke survivors to do rapid movement to reach out a target in different directions to minic the users can quickly reach an objects by hand or make a quick step to restore their balance during a fall incidence to minimize the severity of injury. Arm could reach forward and laterally, and foot could step forward, backward and laterally. We have considered all possible directions with both arms and legs with a total 22 directions. A virtual target was suddenly appeared to indicate different directions on a screen with his affected/unaffected arm/leg. By providing real-time visual feedback, this training was designed to promote both faster movement onset and completion time, as well as larger range-of-motion, to restore the users’ motor functions in balance control and recovery. It was designed to have an easy setup with a depth-sensing camera (Kinect), a mini PC/ laptop and a TV/computer screen, without the need to put any sensor on the users. We hypothesized that RMT could improve stroke survivor’s overall balance and balance recovery performance after training for 20 sessions with the system.

In this study, we aimed to investigate the RMT effectiveness after the 20-session training and compared with conventional balance training (CBT) on survivors with chronic stroke (> 1 year post-stroke. We used “lean-and-release” assessment [38] to provide a simulated falling environment; thus, we can measure the impact of RMT and CBT trainings had on the stroke survivors’ balance recovery reactions. We evaluated their response time on their foot on how they restore their balance during a sudden stimulated fall and their muscle response on 8 major lower limb muscles [i.e. kinematics of the body and electromyography (EMG) of leg muscles] and the clinical assessment scores related to balance. Our study is the only study known to have investigated the effects of both arms and legs with rapid movement training, and used the “lean-and-release” assessment to investigate the reaction and muscle response before and after the training on persons after stroke.

## Methods

### Study design

**Fig. 1:**
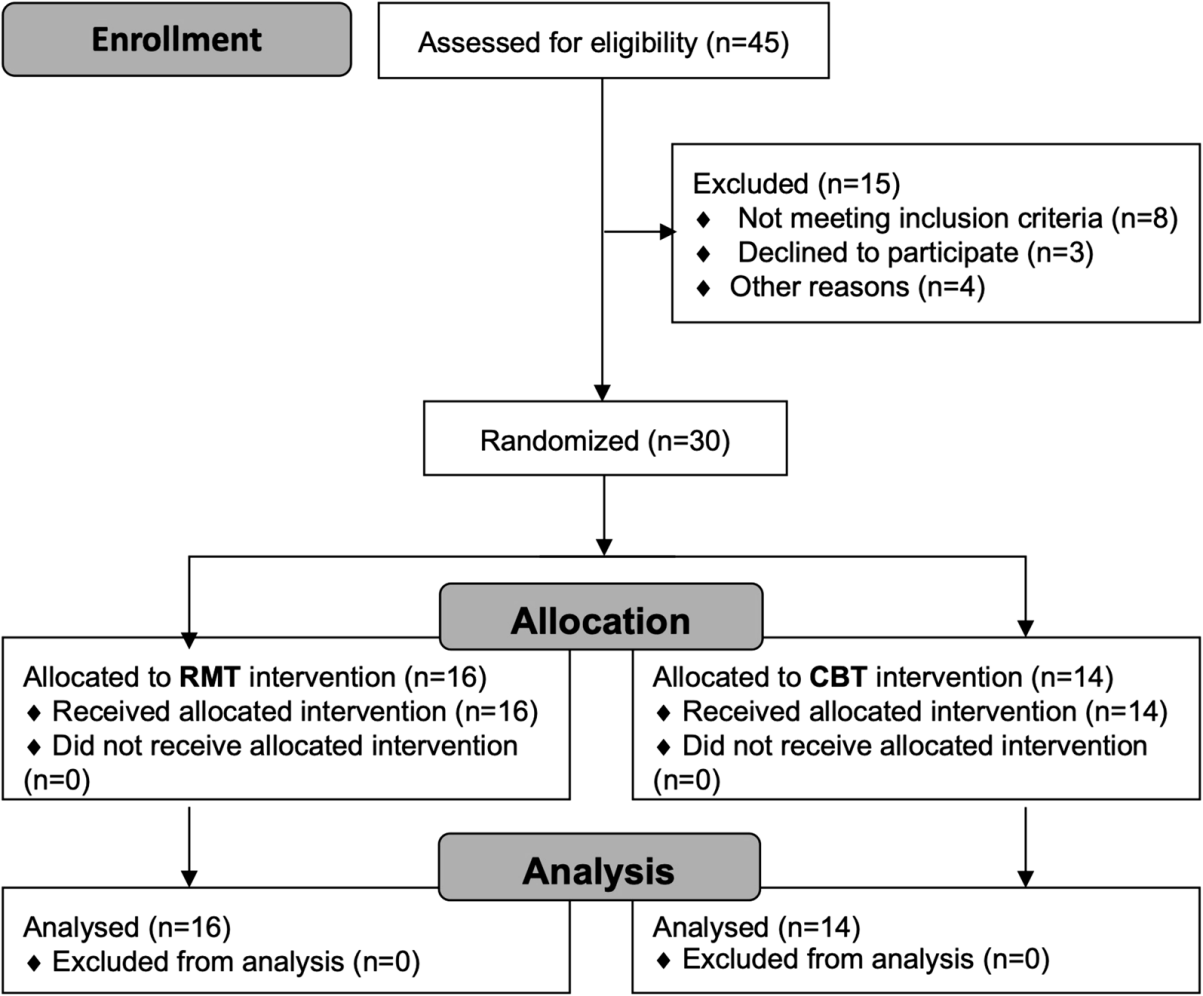
CONSORT flowchart of the randomized controlled trial. *RMT* rapid movement training, *CBT* conventional balance training

This study was an assessor-blind, parallel-group, randomized controlled trial. The study has been approved by The Joint Chinese University of Hong Kong – New Territories East Cluster Research Ethics Committee (No.: 2014.570) and registered at Clinicaltrials.gov (Identifier: NCT03183635). Written informed consents were obtained from each participant. The recruited participants were randomly allocated to either the RMT or CBT group using a random number generator on a computer by an investigator uninvolved in the assessments. The details are depicted in the Consolidated Standards of Reporting Trials (CONSORT) chart shown in Fig. 1. The participants’ profile is shown in Table 1. The participants were assessed before (pre-) and after the training (post-training) by a blinded assessor.

**Table 1:** Demographics and Baseline characteristics for participants analyzed the studies^a^

Variable	RMT Group	CBT Group	*p* value
No. of participants	16	14	
Age (y)	60.6 (5.5)	60.1 (5.8)	0.473^c^
Sex (men:women)	12:4	12:2	0.464^b^
Height (m)	1.65 (0.08)	1.64 (0.07)	0.615
Weight (kg)	65.7 (9.9)	62.8 (10.5)	0.313^c^
Affected side (left:right)	8:8	7:7	1.000^b^
Stroke type (ischemic:hemorrhagic)	11:5	11:3	0.544^b^
Duration of stroke (years)	7.4 (4.1)	6.4 (4.5)	0.334^c^
Fall in the past 3 months (no. of participants)	6/16	5/14	0.919^b^
Mini-Mental-Status-Examination Score (out of 30)	27.8 (2.7)	27.5 (2.7)	0.728^c^
Motivation for training (1–6 Likert Scale)	5.4 (0.5)	5.0 (0.7)	0.179^c^
Activities Balance Confidence Scale (%)	74.2 (11.4)	67.5 (20.0)	0.377^c^
Barthel Index for Activities of Daily Living Scale (out of 20)	19.6 (0.5)	19.3 (1.3)	0.984^c^
Berg Balance Scale (out of 56)	49.1 (1.6)	48.6 (2.6)	0.854^c^
Timed-Up-and-Go (s)	14.7 (3.4)	18.2 (7.57)	0.131^c^
Fugl-Meyer Assessment Score (out of 100)	60.6 (18.2)	58.6 (16.4)	0.667^c^

^a^Values are means (SD) unless otherwise stated

^b^Chi-square test

^c^Mann-Whitney *U* test

Both RMT and CBT groups received twenty training sessions conducted by trainer who were not involved in the assessments. Each session lasted for 60 min, with three training sessions per week over a period of around seven weeks. Assessments for pre- and post-training included “lean-and-release” assessment and clinical score measures of balance confidence, balance, motor functioning, and independent mobility. All assessments were performed on the same day at the Sports Biomechanics Laboratory at the Chinese University of Hong Kong.

### Participants

Potential participants were identified from the Hong Kong Stroke Association. Inclusion criteria were first unilateral stroke with onset > 1 year, age ≥ 50 years old, able to stand independently without aid for at least 15 min, had some level of deficit in balance control, i.e., Berg Balance Scale < 52/56 [9], Snellen visual acuity score of 20/40 [38], and Cantonese Mini-Mental State Examination (CMMSE) ≥ 22/30 (i.e. the cutoff score with 100% sensitivity in identifying cognitively intact individuals using the CMMSE [39]). Exclusion criteria included participation in other training/study, other neurological conditions, cardiovascular condition [6], other serious diseases or conditions (e.g., osteoporosis) [9].

G*Power 3.1 was used to determine the sample size needed to demonstrate significant balance improvement after our training. Effect size (η^2^_p_ = 0.2) was extracted from a similar randomized controlled trial on balance intervention using virtual reality-based stepping exercise for chronic stroke survivors [37]. Using a more conservative estimation of η^2^_p_ = 0.1, sample size calculation for repeated measures ANOVA (2 groups, 2 measurements) determined that a minimum of 26 total participants would be required with α = 0.05 and (1–β) = 0.90. In anticipation of a 10% dropout rate, a total of 30 participants were eventually recruited for this study.

### Rapid movement training (RMT) intervention

The Kinect-based rapid movement training platform system prompted the RMT group participants with a limb (arm or leg) and a direction cue on a screen and tracked the 3D trajectory and timing of their movements. Participants were encouraged to perform arms and legs movement in 22 different directions as quickly and as far as possible [36]. The 22 directions were randomized and were repeated four times. The whole training lasted for an hour.

**Fig. 2:**
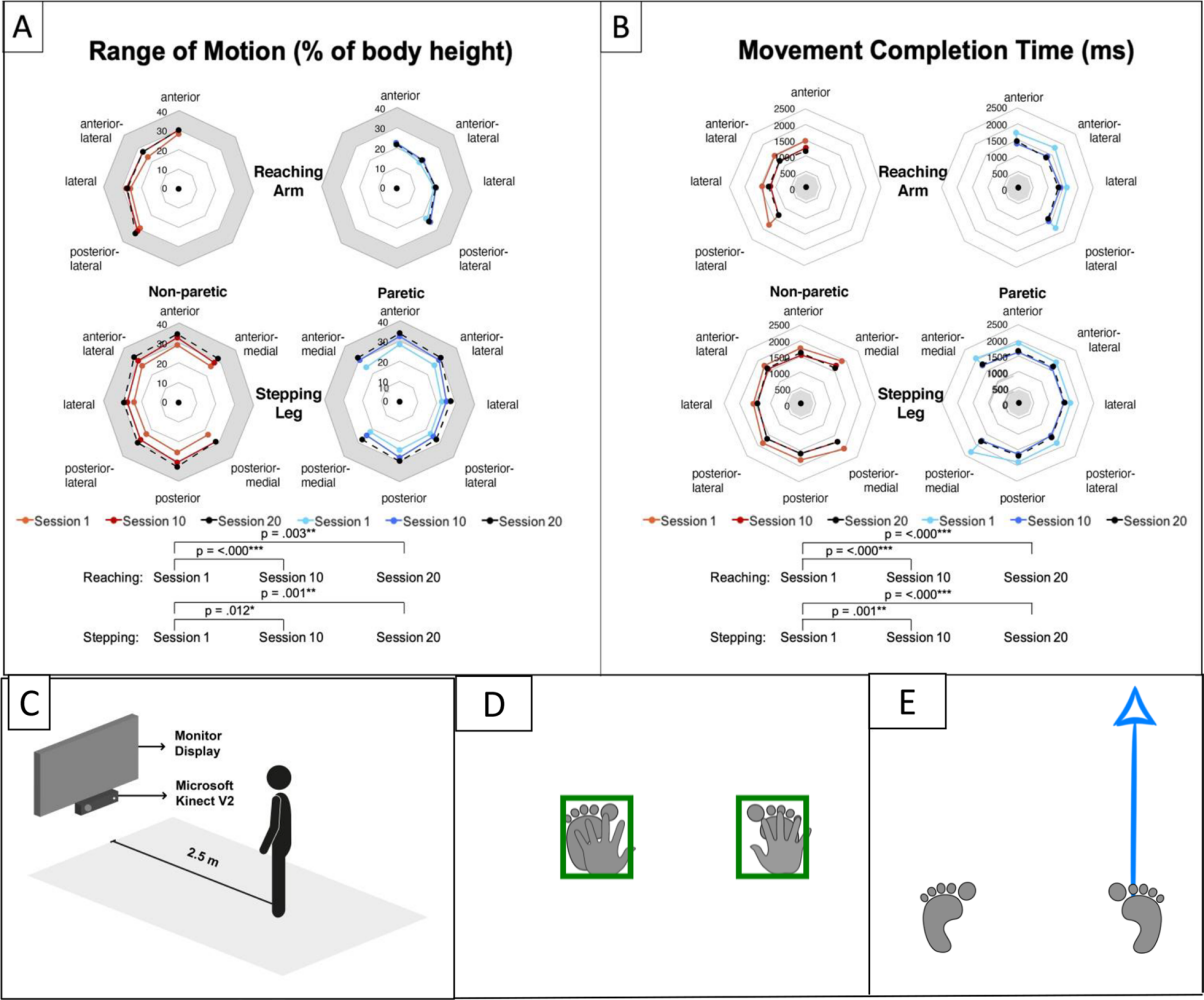
RMT training effects from sessions 1, 10 and 20 on **A** Range-of-motion and **B** Movement Completion Time. The grey colored areas are the target region for the arms and legs for the range-of-motion and the movement completion time. The figure shows the Range-of-motion for both non-paretic and paretic legs increased. Non-paretic and paretic arms and legs became faster in their Movement Completion Time. **C** The RMT system setup. **D** The user needed to stand in an upright position with arms rest on side and feet were placed at shoulder width. The system was real-time show the arms and feet position. The users need to place all of them in the initial positions [Green boxes] **E** This is a sample screen to indicate which limb and which direction need to respond, and this case is Right Foot-Anterior direction

The participants wore safety harness throughout the RMT. The other end of the harness was latched into an omnidirectional movable harness frame. The training setup is illustrated in Fig. 2C. Figure 2D shows the indicator boxes where the subject start and return to before the start of next movement instruction. The participants moved their right or left arm or leg based on the limb icon and direction arrow as in Fig. 2E shown on the training system screen. Participants were instructed to “move as far and as fast as possible.” The participants were asked to move in maximum distance in the horizontal plane as fast as possible for the arms. For the legs, the participants were asked to step as far as possible and as fast as possible while maintaining balance. Wrist joint centers were analyzed for reaching trials and ankle joint centers were analyzed for stepping trials. Reaction time measured when the movement was started and range of motion (ROM) was the final displacement of that intended movement. Reaction time was calculated at the point in which the velocity of the joint equals to five percent of the resultant peak velocity [44]. Movement completion time was calculated at the point in which the velocity of the joint equals to five percent of the resultant peak velocity after the resultant peak velocity has passed. The ROM is the trajectory displacement of how far the joint reached at the completion time. [44]. The range-of-motion, response time and movement completion time of the prompted limb from each trial were collected by the Kinect system, and results from session one, ten, and twenty were analyzed to quantify the progress of training (Fig. 2).

### Conventional balance training (CBT) intervention

The CBT group performed a set of balance exercises that are typically prescribed by physiotherapists from Shatin Hospital Physiotherapy Department to chronic stroke survivors in clinical settings [40]. For the CBT group, the exercises included balance and functional weight-shifting training: sitting-to-standing (using a stool), lateral stepping (walk 3 m back and forth), forward and backward stepping (5 times right leg steps first and 5 times left leg steps first), forward walking for five meters (walk 3 m back and forth, turn right at one end and turn left at one end), stepping up and down (5 times right leg steps first and 5 times left leg steps first), and throwing and catching plastic ball (using a soft volleyball) or small bean bag. The exercises of balance training could effectively improve balance capacity in chronic stroke survivors [17]. These exercises were repeated three times in each training session and each session lasted for an hour.

### Assessments

All participants were evaluated by the same blinded assessor both before and after the intervention using standardized clinical tests and “lean-and-release” assessment. Berg Balance Scale (BBS) and Timed-Up-and-Go Test (TUG) are the primary outcome measures as these were recommended as screening tools for falls and associated with future falls. [23, 24]. Other clinical scores, lean-and-release assessment and EMG measurements are the secondary outcome measures. The clinical scores collected were the BBS for their balance control [23], TUG for gait control which has significant positive relationship with history of falls [24], Fugl-Meyer Assessment of Motor Recovery After Stroke (FMA) for their motor function, Activities specific Balance Confidence Scale (ABC) for their fear of falling, and Barthel Index of Activities of Daily Living (BI) for their functional independence. After the intervention, all participants were asked a question about their motivation of using the RMT/CBT for balance training in their stroke rehabilitation program using a 6-point Likert scale: “Rate your motivation to attend the training program on a scale from 1 to 6, with 6 being very motivated and 1 being no motivation at all?” The results would show whether these two training programs were acceptable by the participants.

The “lean-and-release” assessment was using a balance perturbation system [41]. It mimicked a forward fall situation in laboratory setting to evaluate one’s capacity to restore postural stability when exposed to a balance disturbance. During the assessment, the participant was held in leaning forward position by a horizontal cable behind him/her at the chest level. The participant leaned forward for about 10 degrees with no bending of the hip, arms, and legs, and stood in a standardized foot position (heels are 17 cm apart and 14^*◦*^ angle between the long axes of the feet [42]). Arm and leg muscles were relaxed at the start of each trial as verified by their EMG signal. Behind them, a cable that supported 10% of the participant’s body weight during the forward lean was suddenly released after some random delay of 1–5 s [41]. When the cable was released unexpectedly, the participant was triggered to perform a reactive balance recovery action in a simulated anterior fall independently, and support from the research staff was deemed a failed balance recovery attempt. The participant always wore a safety harness that was latched onto an omnidirectional adjustable harness frame to prevent actual fall from happening. This “lean-and-release” perturbation mechanism has been used in research settings to trigger balance recovery reactions [11, 43].

### Data collection and processing for the “lean-and-release” assessment

The participant’s step displacement to height in percentage (width [medial–lateral direction], length [anterior–posterior direction], and total displacement [the combined displacement of the width and the length] [44], foot movement onset time (MOT), movement completion time (MCT), number of steps, and COM falling velocity (${v}_{COM}$) were extracted from kinematic data recorded by the Bonita 10 Vicon motion capture system (Vicon Motion Systems Ltd, Oxford, UK) with 200 Hz sampling rate. The location of the COM was determined using the Plug-in Gait model of the Vicon system. The kinematic trajectories were low pass filtered (4th order Butterworth 6 Hz cut-off [44]). The participant’s foot off time was recorded by the force plate (Kistler Inc., Amherst, NY, USA). Failed balance recovery attempts (counted as fall) and the number of steps were determined by observing the video camera recording from the Vicon system.

In addition, electromyography (EMG) was used to determine muscle reaction time. Following SENIAM recommendations [45], EMG electrodes were placed bilaterally on the muscle bellies of four major leg muscles: medial gastrocnemius (MG), tibialis anterior (TA), bicep femoris (BF), and rectus femoris (RF) after standard skin preparation procedures [46]. EMG signals from these muscles were sampled at 1500 Hz (Noraxon U.S.A. Inc., Scottsdale, AZ, USA) and was filtered by a bandpass filter (fourth-order, zero-lag Butterworth) in the range of 10–500 Hz to remove the low frequency movement artefact and high frequency noises. Additionally, a 50 Hz notch filter was used to remove unwanted line frequency after power spectrum analysis revealed the presence of 50 Hz electromagnetic interference in our signals [44]. Linear envelopes of the muscle activities were then created by full-wave rectification of the filtered signals, followed by low pass filter (fourth-order, zero-lag Butterworth) at 100 Hz. Muscle reaction time was defined as an increase in muscle activity in the linear envelope (i.e. > 2SD above the mean signal 1 s prior to perturbation onset) for at least 30 ms [38, 44].

### Statistical analysis

For the RMT group, statistical analyses for reaching and stepping range-of-motion and movement completion time collected from the first, tenth, and twentieth sessions were done using repeated measures analysis of variance (RM-ANOVA) to quantify their progress of improvement. Range-of-motion and movement completion time were continuous and normally distributed. Time, Side (Paretic vs Non-paretic) and Direction main effects, as well as their interaction effects were analyzed.

For the assessments, dependent variables were analyzed and tested for normal distribution with Shapiro–Wilk test. Baseline characteristics of participants were compared between RMT and CBT groups using independent t-test for normally distributed continuous variables, Mann–Whitney U test for non-normally distributed continuous variables or ordinal variables, and chi-square test for categorical data. For non-normally distributed and ordinal dependent variables (e.g. all the clinical scores), the Wilcoxon Signed Rank test was used to compare Time main effect and the Mann–Whitney U test was used to compare Group main effect, as well as Group × Time interaction effect using the difference between pre- and post-training values. On the other hand, RM-ANOVA was conducted on all kinematic and EMG dependent variables collected from the “lean-and-release” assessment to find the Time main effect, Group main effect, and Group × Time interaction effect since they were all normally distributed [47].

All statistical analysis was processed with SPSS Statistics version 25.0 (*IBM Corp.*, Armonk, NY, USA) with alpha level set at 0.05.

## Results

A total of forty-five potential participants were screened in 2017 and 2018, but only thirty were eventually recruited based on our inclusion/exclusion criteria. Although the chance of being allocated to either group was the same at the subject randomization process, eventually sixteen participants were randomized to the RMT arm and fourteen to the CBT arm. Two participants, one from each group, were excluded in the “lean-and-release” assessment analysis due to their inability to execute balance-recovery reactions in any trial. Demographic and clinical characteristics of participants were shown in Table 1. There was no difference in the clinical profile between the two groups. The RMT and CBT treatments were well accepted by our participants as depicted from their high motivation to attend training sessions in the 6-point Likert scale (6 is very motivated): 5.4 (SD = 0.5) for RMT and 5.0 (SD = 0.7) for CBT.

### Progressive improvement of RMT

Figure 2A, B illustrates the RMT training effects on range-of motion (% of body height) and movement completion time as collected by the Kinect system in session ten and twenty compared with session one. Only Time main effect was significant (P < 0.05). Participants in RMT group showed improvement in movement completion time (faster) and range-of-motion (larger) for both paretic and non-paretic side reaching arms and stepping legs between the first, tenth, and last (i.e. twentieth) training session. For range-of-motion, the non-paretic arm and both legs could often reach 30% body height by the last training session, which was typically required to re-establish postural stability in successful change-in-support reactions in healthy adults [48, 49].

### Clinical scores results

As summarized in Table 2, both groups showed significant improvements in balance and motor function as indicated by improvement in BBS (primary outcome; *P* < 0.001) and FMA (*P* = 0.01) clinical scores, respectively, despite the lack of significant main Group effect in all clinical score analyses. There appeared to be a trend of improvement in gait control with moderately faster TUG results for both groups post-training (*P* = 0.07). No significance differences were found in ABC and BI. For the RMT group only, the participants showed significant improvements in BBS (*P* = 0.001), TUG (*P* = 0.011), and FMA (*P* = 0.015). On the other hand, the CBT group showed significant improvements only in BBS (*P* = 0.005).

**Table 2:** Results from clinical scores and “lean-and-release” assessment including the COM velocity

Outcome measures	Rapid Movement Training Group (RMT)			Conventional Balance Training Group (CBT)			*P*	
	Pre	Post	P	Pre	Post	*P*	Time	Group x Time
Primary outcome measures								
BBS	49.13 (1.63)	52.75 (2.46)	0.001**	48.64 (2.56)	52.00 (2.96)	0.005**	< 0.001***	0.886
TUG (s)	14.66 (3.42)	12.62 (2.54)	0.011*	18.16 (7.57)	17.65 (5.79)	0.950	0.070	0.085
Secondary outcome measures								
FMA	60.63 (18.20)	65.19 (17.14)	0.015*	58.64 (16.43)	60.43 (17.34)	0.257	0.010**	0.790
ABC (%)	74.22 (11.41)	74.59 (19.07)	0.943	67.53 (20.00)	70.62 (17.84)	0.300	0.147	0.697
BI	19.63 (0.50)	19.69 (0.60)	0.669	19.29 (1.33)	19.21 (1.85)	0.856	1.000	0.637
Step displacement (% of height)	28.33 (9.69)	29.66 (9.08)	0.166	30.55 (8.94)	28.92 (9.11)	0.039*	0.798	0.018*
Step length (% of height)	28.19 (9.64)	29.48 (9.01)	0.176	30.40 (8.95)	28.78 (9.10)	0.043*	0.778	0.020*
Step width (% of height)	2.20 (1.96)	2.72 (2.10)	0.295	2.17 (1.87)	2.44 (1.47)	0.349	0.181	0.666
Movement onset time (s)	229.60 (74.25)	218.83 (65.26)	0.108	228.67 (91.71)	221.85 (68.80)	0.440	0.103	0.707
Movement completion time (s)	496.62 (137.63)	494.98 (130.88)	0.800	493.38 (149.14)	481.11 (135.84)	0.346	0.312	0.437
Number of steps	2.37 (1.25)	2.39 (1.34)	0.904	2.83 (1.47)	2.85 (1.44)	0.967	0.925	0.988
${\text{v}}_{\text{COM}}$ at MOT (mm/s)	180.73 (108.57)	201.56 (107.32)	0.289	190.72 (98.50)	177.81 (90.70)	0.335	0.740	0.164
${\mathrm{v}}_{\mathrm{COM}}$ at MCT (mm/s)	763.89 (240.19)	773.80 (229.10)	0.750	760.23 (224.19)	783.86 (230.40)	0.394	0.422	0.741

*BBS* Berg Balance Scale, *FMA* Fugl-Meyer Assessment, *TUG* Timed-Up-and-Go, *ABC* Activities Balance Confidence Scale, *BI* Barthel Index for Activities of Daily Living, *MOT* movement onset time, *MCT* movement completion time, ${\mathrm{v}}_{\mathrm{COM}}$, velocity of center of mass

Clinical scores such as BBS, TUG, FMA, ABC and BI (RMT n = 16, CBT n = 14) were analyzed using Mann–Whitney U test (Group × Time) and Wilcoxon Singed Rank Test (Time). “Lean-and-release” assessment results such as spatiotemporal data (RMT n = 15, CBT n = 13) were analyzed using repeated measures ANOVA

The bold indicate statistically significant results. *P < 0.05; **P < 0.01, ***P < 0.001

### “Lean-and-release” assessment – kinematic results

Table 2 also shows the kinematic data from the “lean-and-release” assessment in pre- and post-training. In general, subjects with chronic stroke preferred to step with the non-paretic leg more than the paretic leg (non-paretic leg to make the first step was 88.8% of the trials in both groups in pre-training and 89.9% in post-training). Both the RMT and CBT group did not change their preference of using the non-paretic leg for stepping during simulated falls after the intervention; therefore, minimizing the potential confounding effect of using legs with different ability in the two assessments.

Significant Group × Time interaction effect was observed for step overall displacement (*P* = 0.018) and step length (*P* = 0.020). Post-hoc analyses (i.e. separate RM-ANOVA for the RMT and CBT groups) revealed that step overall displacement (*P* = 0.166) and step length (*P* = 0.176) increased post-training for the RMT group only. In the CBT group, step overall displacement (*P* = 0.039) and step length (*P* = 0.043) decreased. No statistical significant changes were observed for movement onset time, movement completion time, number of steps and velocity of COM with neither group.

### “Lean-and-release” assessment – EMG results

**Fig. 3:**
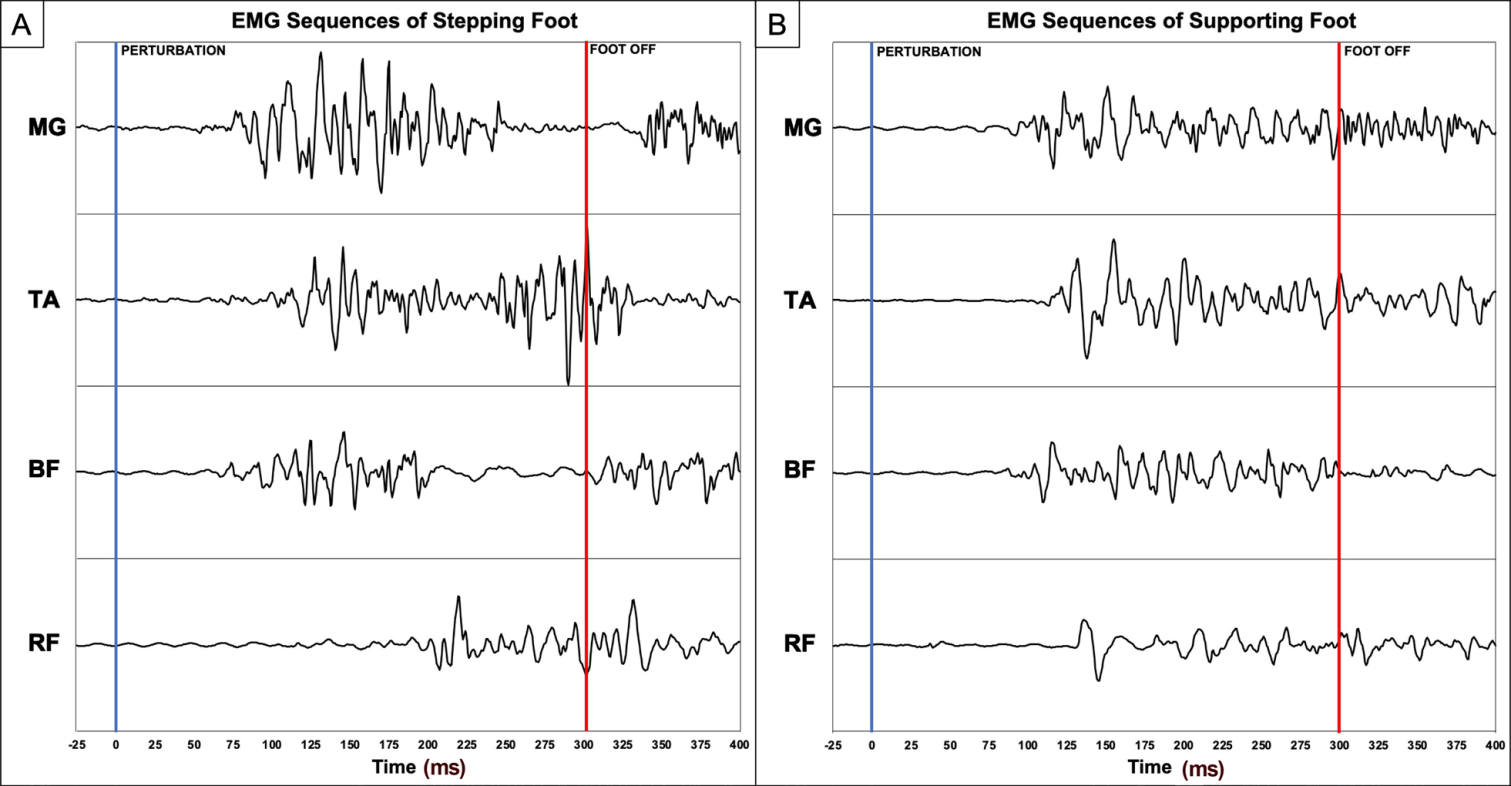
EMG Activation Sequences of **A** Stepping Legs and **B** Supporting Legs. *MG* medial gastrocnemius, *TA* tibialis anterior, *BF* biceps femoris, *RF* rectus femoris

**Fig. 4:**
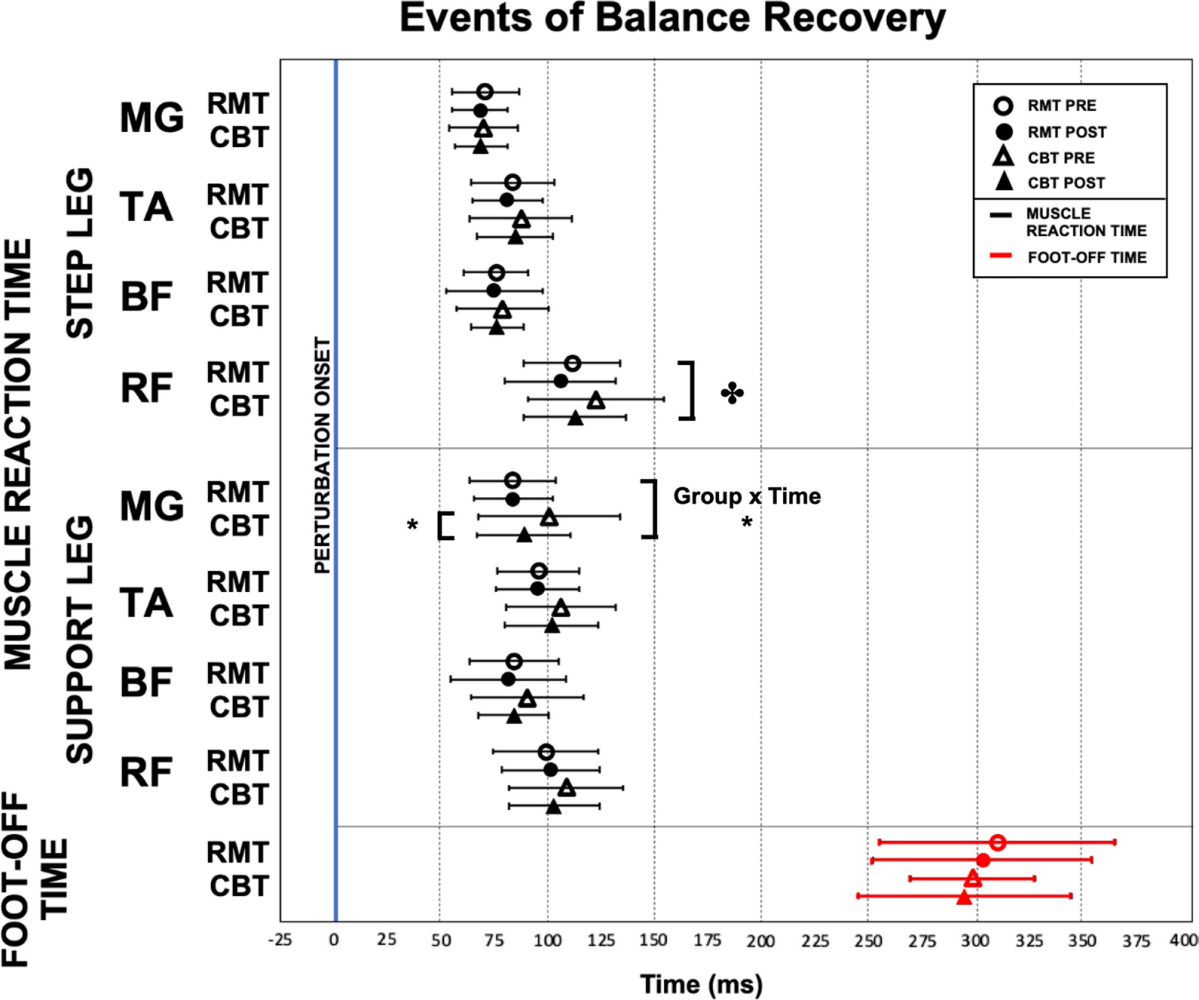
Image showing muscle reaction time for stepping and support leg during “lean-and-release” fall assessment. RMT, Rapid Movement Training; CBT, Conventional Balance Training; MG, medial gastrocnemius; TA, tibialis anterior; BF, biceps femoris; RF, rectus femoris (*P < 0.05; **✤**Time effects)

Figure 3 illustrates a sample of the EMG signal collected from the “lean-and-release” assessment that showed the muscle activation patterns for the stepping and supporting leg on an individual subject’s EMG signals and Fig. 4 is the group analysis results. Most of the muscle reaction times for both the stepping and supporting legs remained similar before and after either mode of training. Significant Time main effect was observed in the rectus femoris of the stepping leg (*P* = 0.036), as both groups were able to activate that muscle faster in their post-training assessments. Significant Group × Time interaction effect (*P* = 0.022) in the supporting leg medial gastrocnemius revealed that only the CBT group had significantly faster reaction time (*P* = 0.016) for that muscle after training.

## Discussion

The current study supports our hypothesis that RMT can result in improved overall balance in chronic stroke survivors. As previous studies have shown, perturbation [50, 51] or agility-based training [52], like our RMT, can improve automatic postural responses and reactive stepping performance. Chronic stroke survivors in RMT group significantly improved their BBS, TUG, and FMA score after 20-sessions training. Improvements in BBS, TUG, and FMA in this paper show that RMT and CBT improved overall gross balance and reduction of fall risk. Chronic stroke survivors in both groups also became quicker in their stepping rectus femoris reaction time. Rectus femoris muscle is a part of the quadriceps group. It is a biarticular muscle that crosses over the hip and knee joints, contributing to both hip flexion and knee extension. In the balance recovery reaction, the rectus femoris activity occurs during the loading as well as pre- and initial swing phases of the stepping leg [53]. However, the faster activation of rectus femoris did not lead to significantly faster movement onset time nor movement completion time in our study. The stroke survivors had difficulty in initiating and executing successful reactive stepping responses with their paretic leg, which are shown in previous studies [51, 54] and we also observed this before the 20-session training. Despite training both the paretic and non-paretic limb equally for 20 sessions, participants in the RMT group did not change their stepping leg preference after the training.

Marigold and colleagues showed that the agility training (i.e., including activities such as tandem stance, rapid knee raise while standing, and others) has decreased the stroke survivors’ prevalence of fall incident compared to the traditional training group [9]. A study from Gray’s research team also found that exercises addressed explicitly for improving movement speed can be beneficial for movement velocity for survivors with mild to moderate stroke severity [16]. CBT training of this study was involved with more task-oriented functional training including throwing and catching plastic ball or small bean bag, and sitting-to-standing. Previous studies have shown that the conventional training alone would improve the balance in chronic stroke patients [55]. RMT, on its own, was more specifically focused on improving reaction time and range-of-motion tackles different aspects of balance rehabilitation. Figure 2 shows that RMT stroke participants improved their reaction time and range-of-motion throughout the training sessions. The spatiotemporal measurements captured by RMT are valuable information for clinicians to track patients’ progress more objectively.

We should acknowledge several limitations of the study. Firstly, our results were based on a small sample size that was taken from the chronic stroke population who are functioning well in community settings (i.e. able to stand and walk independently with/without supervision), therefore the results in this study may not be applicable to the other stages of stroke (acute and subacute) or lower functioning chronic stroke survivors in long term care [50]. The feasibility of RMT for acute and subacute stroke survivors need to be confirmed in further studies. Secondly, our “lean-and-release” system was only able to trigger forward falls, and participants might become habituated during the assessment and could anticipate when they were going to fall. This unidirectional nature of the “lean-and-release” assessment limited our dependent variables observation only to anterior stepping despite the multiple directions trained in the RMT.

Further study may also be needed to evaluate the effects of RMT on the execution of balance recovery reactions in other directions other than anterior stepping by using a moving platform [44]. It would also be interesting to investigate whether intensive training on the paretic leg using RMT can encourage stroke survivors to use the paretic leg more often in balance recovery, and whether such training will lead to functional improvement for balance control [54]. Subacute stroke patients might also benefit from the RMT, and studies had shown long-term effects with electromechanical gait training system on subacute stroke rehabilitation [56]. In addition, a survey to collect clinical professionals’ feedback in the future study involving larger sample size in hospital or clinical setting is needed to evaluate its practical use in healthcare setting. Future investigations on combining strength training and RMT can also be explored to see if the faster muscle reaction time can translate into functional outcomes with improved muscle strengths [57]. Difficulty level can be adjusted throughout the training period to keep the task challenging and stimulating.

## Conclusions

The Kinect-based RMT system provided real-time feedback to promote faster arms reaching and legs stepping action toward targets in multiple directions. Twenty sessions of training of RMT has beneficial effects for the chronic stroke survivors in terms of overall balance performance, motor functioning, and fall risk as reflected in the primary outcomes BBS and TUG as well as the FMA and faster stepping rectus femoris reaction time. The results showing that RMT was as effective as conventional balance training rehabilitation. Our findings provide support for introducing RMT for balance training in stroke rehabilitation and has the potential to be applied for home-based telerehabilitation.

## Data Availability

All data generated or analysed during this study are included in this published article [and its supplementary information files]. Information on this clinical trial can be found at Clinicaltrials.gov (https://clinicaltrials.gov/ct2/show/NCT03183635, NCT03183635).
